# Serial echocardiographic evaluation of COVID-19 patients without prior history of structural heart disease: a 1-year follow-up CRACoV-HHS study

**DOI:** 10.3389/fcvm.2023.1230669

**Published:** 2023-09-13

**Authors:** Agnieszka Olszanecka, Wiktoria Wojciechowska, Agnieszka Bednarek, Piotr Kusak, Barbara Wizner, Michał Terlecki, Katarzyna Stolarz-Skrzypek, Marek Klocek, Tomasz Drożdż, Krzysztof Sładek, Monika Bociąga-Jasik, Aleksander Garlicki, Krzysztof Rewiuk, Andrzej Matyja, Maciej Małecki, Wojciech Sydor, Marcin Krzanowski, Tomasz Grodzicki, Marek Rajzer

**Affiliations:** ^1^ 1st Department of Cardiology, Interventional Electrocardiology and Arterial Hypertension, Jagiellonian University Medical College, Kraków, Poland; ^2^ University Hospital in Kraków, Kraków, Poland; ^3^ Department of Internal Diseases and Geriatrics, Jagiellonian University Medical College, Kraków, Poland; ^4^ Department of Pulmonology and Allergology, Jagiellonian University Medical College, Kraków, Poland; ^5^ Department of Infectious and Tropical Diseases, Jagiellonian University Medical College, Kraków, Poland; ^6^ Department of General, Oncological, Metabolic, and Emergency Surgery, Jagiellonian University Medical College, Kraków, Poland; ^7^ Department of Metabolic Diseases and Diabetology, Jagiellonian University Medical College, Kraków, Poland; ^8^ Center for Innovative Therapies, Clinical Research Coordination Center, University Hospital in Kraków, Kraków, Poland; ^9^ Department of Rheumatology and Immunology, Jagiellonian University Medical College, Kraków, Poland; ^10^ Department of Nephrology and Dialysis, Jagiellonian University Medical College, Kraków, Poland

**Keywords:** COVID-19, echocardiography, myocardial oedema, SARS-CoV2, troponin, NT-proBNP

## Abstract

**Background:**

It is a well-known fact that COVID-19 affects the cardiovascular system by exacerbating heart failure in patients with preexisting conditions. However, there is a poor insight into the cardiovascular involvement and sequelae in patients without preexisting conditions. The aim of the study is to analyse the influence of COVID-19 on cardiac performance in patients without prior history of structural heart disease. The study is part of the CRACoV project, which includes a prospective design and a 12-month follow-up period.

**Material and methods:**

The study included 229 patients hospitalised with a diagnosis of COVID-19 (median age of 59 years, 81 were women). A standard clinical assessment and laboratory tests were performed in all participants. An extended echocardiographic image acquisition was performed at baseline and at a 3-, 6-, and 12-month follow-up. All analyses were performed off-line. A series of echocardiographic parameters was compared using repeated measures or Friedman analysis of variance.

**Results:**

In all subjects, the left ventricular (LV) ejection fraction at baseline was preserved [63.0%; Q1:Q3 (60.0–66.0)]. Elevated levels of high-sensitivity cardiac troponin T were detected in 21.3% of the patients, and elevated NT-proBNP levels were detected in 55.8%. At the 1-year follow-up, no significant changes were observed in the LV diameter and volume (LV 48.0 ± 5.2 vs. 47.8 ± 4.8 mm, *p* = 0.08), while a significant improvement of the parameters in the biventricular strain was observed (LV −19.1 ± 3.3% vs. −19.7 ± 2.5%, *p* = 0.01, and right ventricular −19.9 ± 4.5% vs. −23.2 ± 4.9%, *p* = 0.002). In addition, a decrease in the LV wall thickness was also observed (interventricular septum 10.4 ± 1.6 vs. 9.7 ± 2.0 mm, *p* < 0.001; LV posterior wall 9.8 ± 1.4 vs. 9.1 ± 1.5 mm, *p* < 0.001).

**Conclusions:**

In an acute phase of COVID-19, the elevation of cardiac biomarkers in patients with normal left ventricular ejection fraction is a frequent occurrence; however, it does not translate into clinically significant cardiac dysfunction after 1 year. The serial echocardiographic evaluations conducted in patients without preexisting structural heart disease demonstrate an overall trend towards an improved cardiac function and a reduced myocardial thickening at 1-year follow-up. This suggests that the acute cardiac consequences of COVID-19 are associated with systemic inflammation and haemodynamic stress in patients without preexisting conditions.

## 1. Introduction

The COVID-19 pandemic represented unprecedented challenges for medical professionals worldwide. For the first time in the modern history, we witnessed, observed, and examined a highly infectious disease causing respiratory inflammation with multiorgan involvement for a large girth of the global population but also presented numerous possibilities for the application of innovative technology to describe the disease and its epidemiology, transmission, onset and pathogenesis, and prognostic biomarkers. The first observations of patients with COVID-19 indicated that the factors of unfavourable prognosis are age and the coexistence of cardiovascular diseases, including hypertension, diabetes, and chronic kidney disease (1, 2). Considering the biology of the SARS-CoV2 virus that uses the transmembrane angiotensin-converting enzyme type 2 (ACE2) present on cells of various organs (lungs: type II pneumocytes, heart, intestinal epithelium, kidneys, Leydig cells) as well as on the endothelium to enter the cell, the probability of multiorgan damage in the course of SARS-CoV2 infection was predictable (3). Increased markers of myocardial necrosis (troponin) and heart failure (NT-proBNP) have been shown to be associated with an increased risk of death in COVID-19 patients (1, 2, 4–7). Moreover, studies from the initial outbreak in Wuhan reported that a newly diagnosed heart failure was found in 23% of all patients hospitalised with COVID-19, and it was found in 52% of the non-survivors (4). In a European study including patients with COVID-19 admitted to the intensive care unit (ICU), an abnormal cardiac function in echocardiography was detected in 34% of participants (8). Alarmingly high rate of persistent myocardial injury in 60% of the patients at 71 days post-COVID-19 was reported in a follow-up cardiac magnetic resonance (CMR) study from Germany (9). However, the interpretation of these data is hampered by the heterogeneous population under study, variable time of follow-up, and associated comorbidities and risk factors. In fact, preexisting heart disease may overlap on the COVID-19-related changes in patients undergoing clinically indicated echocardiography as well as in patients studied with CMR.

Nevertheless, early observations with regard to cardiac involvement in COVID-19 raised the question about its long-term consequences on the circulatory system. Special concern was related to the possible development of heart failure resulting from systolic dysfunction of the left or right ventricle and pulmonary hypertension as a complication of lung fibrosis secondary to the infection.

There is a poor insight into the cardiovascular involvement and sequelae in those with no preexisting conditions. We performed a systematic and comprehensive serial echocardiographic evaluation of patients hospitalised with COVID-19. The aim of the study was to analyse cardiac performance in subjects without prior history of structural heart disease in relation to inflammatory markers and clinical outcomes at baseline and during the 12 months of follow-up.

## 2. Material and methods

Our study is a part of the CRACoV-HHS project that aims at integrating basic and clinical research to better understand the pathomechanism, the course of infection, and the prognosis, including early and long-term physical and mental health complications associated with COVID-19, with details published elsewhere (10). The study was approved by the local Bioethics Committee, and informed consent was obtained from participants before enrolment.

Of the 498 patients with COVID-19 included in the CRACoV study, 229 patients were qualified to be included in the echocardiographic sub-study. The primary inclusion criteria consisted of the following: (1) informed consent to participate in the study, (2) confirmed COVID-19 infection (positive RT-PCR or antigen test), and (3) age of ≥18 and <75 years. The patients were recruited between 8 January 2021 and 30 April 2021. Patients without prior history of structural heart disease were eligible for the study. The exclusion criteria were the following: (1) a prior diagnosis of left ventricular systolic dysfunction with an ejection fraction (EF) of <40%, (2) a prior diagnosis of severe valvular heart disease, (3) a history of an atherosclerotic cardiovascular event within 6 months prior to inclusion in the study (i.e., stroke, myocardial infarction, angioplasty of coronary or peripheral arteries, coronary artery bypass grafting), (4) chronic kidney disease with eGFR of <30 ml/min/1.73 m^2^ at admission, (5) active cancer, and (6) chronic inflammatory disease.

In all patients included in the study, a comprehensive medical history was taken according to a standardised questionnaire, and a physical examination was performed. The severity of the disease on the admission was categorised into asymptomatic, mild, moderate, severe, and critical according to the COVID-19 Treatment Guidelines provided by the National Institutes of Health (11).

### 2.1. Laboratory diagnostics

Blood samples were collected on the first (H1) and seventh day of hospitalisation (H7), and cardiac and inflammatory markers were assessed [high-sensitivity cardiac troponin T (hs-cTnT) measured by the enzyme-linked immuno-culture assay (ELICA) using a Cobas Pro device (Roche Diagnostics GmbH, Mannheim, Germany); NT-proBNP measured by ELICA using a Cobas Pro device (Roche Diagnostics); high-sensitivity C-reactive protein (hs-CRP) measured by nephelometry using a Siemens BN II device (Siemens Healthineers, Erlangen, Germany); D-dimer measured by coagulometry using a Siemens Atellica COAG 360 device (Siemens Healthineers)].

### 2.2. Echocardiography

After the inclusion criteria were met, a bedside echocardiography was performed within the first 72 h after admission in 185 patients hospitalised in the New University Hospital. The bedside echocardiography was performed using the Vivid IQ device (GE Ultrasound, Horten, Norway). Patients hospitalised in the Temporary COVID Ward (*n *=* *44) had their first echocardiographic examination performed at the first check-up, which was 4 weeks after discharge from the hospital.

**Figure 1:**
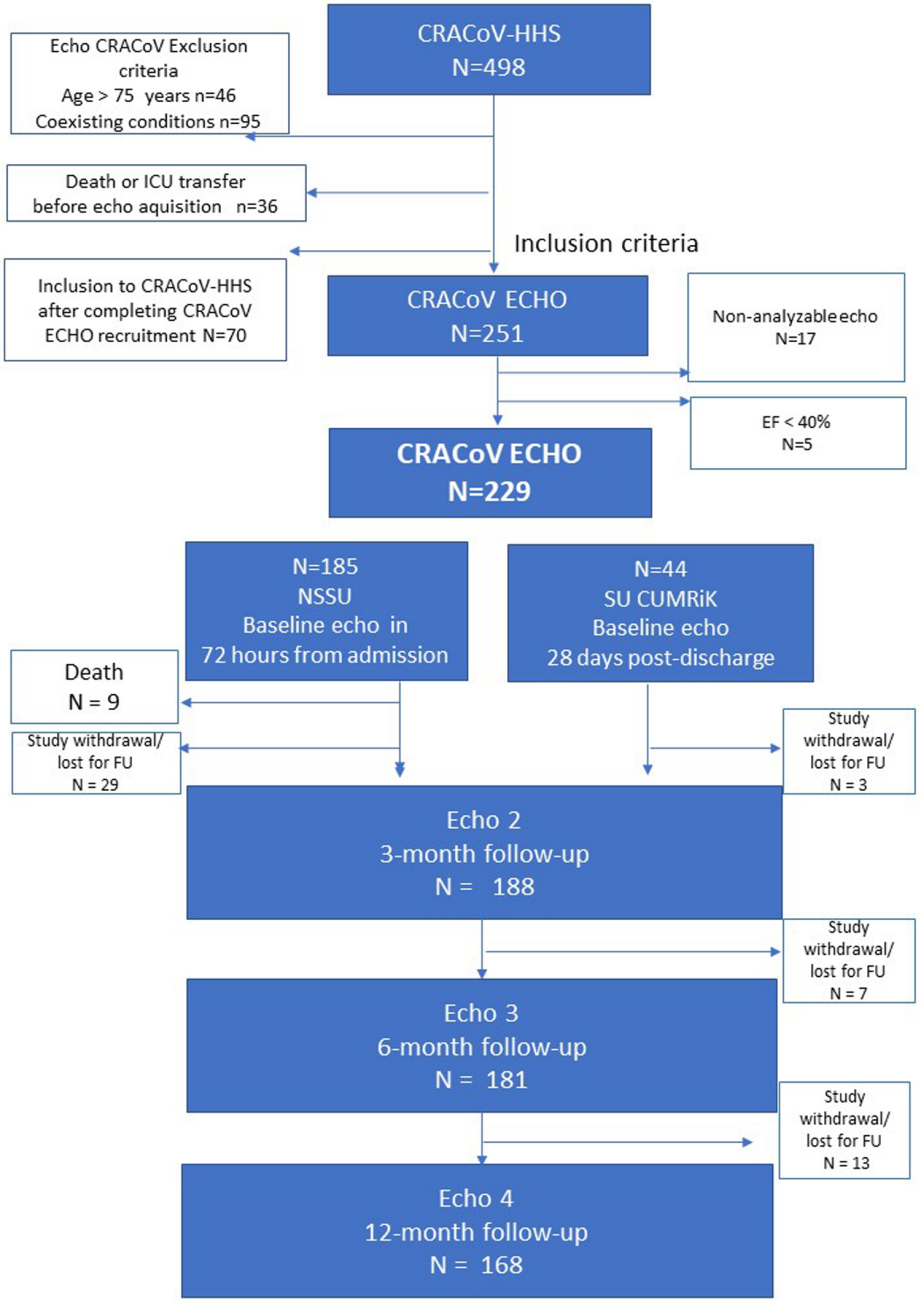
Study flowchart.

Two experienced sonographers performed the image acquisition according to a standardised predefined protocol by recording three heart cycles in each of the protocol sequences. The examinations were archived in the ViewPoint 6 system (ViewPoint, GE Medical Systems, Horten, Norway). The study analyses were performed off-line using an EchoPack v204 workstation integrated with the ViewPoint system. All follow-up examinations were performed using the GE Vivid E95 device (GE Ultrasound, Horten, Norway). All patients underwent a follow-up examination after 3, 6, and 12 months. Figure 1 presents a flowchart of the study.

Trained investigators performed the off-line image analysis of the echocardiographic examinations, and they were blinded to all clinical information including the order of the study for each patient. However, they were not blinded to the specific date of the examination. All conventional measurements of the chamber size and volumes were performed following the recommendations of the guidelines (12, 13).

The left ventricle (LV) EF was calculated with the use of the Simpson's biplane method. Left ventricular mass (LVM) was calculated according to the Devereux cube formula

$$\left(0.8\;[1.04\;(\mathrm{IVSTd}\;+\;\mathrm{LVIDD}\;+\;\mathrm{PWTd}{)}^{3}\;\text{–}(\mathrm{LVIDd}{)}^{3}]\;+\;0.6g\right)$$

and indexed to the body surface area. The relative wall thickness (RWT) was calculated as two times posterior wall thickness divided by LV internal diameter at the end-diastole.

As an estimate of the global right ventricular (RV) systolic function, the fractional area change (FAC) was measured. In the images optimised on maximising the right ventricle, the border of the endocardium was traced in the end-systole and end-diastole, and the difference in the end-diastolic area and end-systolic area divided by the end-diastolic area was calculated. The function of RV longitudinal fibres was measured by calculating the tricuspid annular plane systolic excursion (TAPSE) in M-mode of the apical four-chamber projection, as well as by the systolic velocity of the tricuspid annulus (S’) assessed by pulsed tissue Doppler.

The right atrial area and volume (RAV) was calculated according to the single disk summation technique. Left atrial volume (LAV) was measured based on the biplane disk summation technique and further indexed to body surface area (LAVi).

Using the continuity equation with measurement of the left ventricular outflow tract diameter (LVOT) and pulsed-wave Doppler velocity time integral, the left ventricular stroke volume (SV) and cardiac output (CO) were calculated.

The maximal velocity of tricuspid regurgitation (TR Vmax) was measured by the continuous wave Doppler across the tricuspid valve, and by following the guidelines, the echocardiographic probability of pulmonary hypertension was calculated, integrating data from other measurements obtained (14).

Mitral inflow velocities were recorded with pulsed-wave Doppler sonography to assess the left ventricular diastolic function. Three consecutive cardiac cycles were averaged to measure the peak velocities reached in early diastole (E-wave) and after atrial contraction (A-wave), and calculate the E/A ratio. Colour-coded tissue Doppler images were acquired for lateral and septal segments of the mitral annulus. The sample volume was placed at the junction of the LV wall with the mitral annulus of the septal and lateral myocardial segments from the four-chamber view. The peak velocities during systole (*S’*), early diastole (*E’*), and late diastole (*A’*) were measured. The final value represented the average of two sites. The ratio of early mitral inflow and mitral annulus velocities (*E*/*E*’) was calculated.

According to the latest guidelines, left ventricular diastolic dysfunction (LVDD) assessment was based on four parameters: E′, E/E′ ratio, LAVI, and TR V max (13). Their cutoff values are as follows: septal E’ of <7 cm/s, lateral E’ of <10 cm/s, E/E’ of >14, LAVI of >34 ml/m^2^, and TR V max of >2.8 m/s. LVDD was diagnosed if three or more parameters were abnormal, diastolic function was indeterminate if two were abnormal, and diastolic function was considered normal if three were normal.

A two-dimensional speckle tracking technique and dedicated semi-automated software were employed to calculate left and right ventricular and left atrial strain (LAS). Projections with the highest available frame rate were used. The measurement of the left ventricular global longitudinal strain (GLS) was performed in the three apical long-axis views. The region of interest (ROI) was manually traced. From each projection, six regions were included into a global model of the LV. GLS was calculated as the average of all segments included by an automated software function.

The right ventricular longitudinal strain (RVLS) was measured in an apical four-chamber projection optimised for a view of the RV. The RV free wall was divided into three segments. The peak systolic strain was calculated separately for the RV free wall and RV including septal deformation.

LAS was calculated from the apical four- and two-chamber projections. The ROI was defined with landmarks at the endocardial border on both sides of the mitral annulus and one landmark at the atrial roof. The region of interest provided by the system was manually edited as required. Then, the system automatically tracked speckles inside the ROI and provided strain measurements with zero strain defined at the left ventricular end-diastole (R-wave of the electrocardiogram). Based on the cardiac cycle, three components of LAS were defined: reservoir strain (LAS-r), conduit phase strain (LAS-cd), and contractile strain (LAS-ct).

### 2.3. Statistical analysis

Individual characteristics are presented as numbers and percentages for categorical variables, and depending on the variable distribution, normally or non-normally distributed variables are presented as means with standard deviations (SD) or medians with 25th–75th percentiles, respectively.

The Shapiro–Wilk test was used to verify the normality. The differences between independent groups, such as baseline severity of the disease or in-hospital outcome, were compared with Student’s *t*-test or the Mann–Whitney *U* test for non-normally distributed continuous variables. The categorical variables were compared between independent groups based on chi-square testing or Fisher's exact test.

Paired *t*-test for continuous variables with normal distribution and Wilcoxon rank-sum test for variables with non-normal distribution were used to analyse the changes between repeated measurements taken in consecutive follow-up time points compared with baseline points. A series of echocardiographic parameters was compared using repeated measures or Friedman analysis of variance (ANOVA) depending on whether parametric assumptions are met. Wilcoxon rank-sum test for pairwise comparisons was used following Friedman's analysis of variance. Correlation coefficients between two variables were calculated using Spearman's correlation test.

A multiple logistic regression was used to evaluate the risk factors associated with unfavourable COVID-19 prognosis defined as composite end-point consisting of in-hospital death, intensive care unit admission, or respiratory failure requiring high-flow oxygen therapy. To evaluate the discriminative performance of the logistic model, the area under the receiver operating characteristic (ROC) curve was calculated, comparing the actual outcome to the outcome predicted by the model.

Intra- and interobserver variability were calculated using the weighted Cohen's Kappa and the intraclass correlation coefficient (ICC). All analyses were performed using STATISTICA version 13 software (2017; TIBCO Software Inc., Palo Alto, CA, USA). Two-sided tests were used, and *p* < 0.05 was set as the level of significance.

## 3. Results

### 3.1. Baseline demographic and clinical characteristics

The echocardiographic arm of the CRACoV-HHS project included 148 men (65%) and 81 women (35%) in the study, and the median age of the population under study was 59 years (48–67). The characteristics of the study population are presented in Table 1. The majority of patients were hospitalised due to a symptomatic COVID-19; 11 patients (4.8%) were admitted with an alternative primary admitting diagnosis. The clinical spectrum of infection ranged from asymptomatic in six patients (2.6%), mild in 20 patients (8.7%), moderate in 83 patients (36.2%) to severe in 120 patients (52.4%) and critical in one patient (0.4%). Progressing from initially non-severe disease defined at admission to severe disease was observed in 39 (17.0%) of patients during hospitalisation. Eight patients (3.4%) required admission to an ICU, the majority (70.9%) required supplemental oxygen therapy with 22 patients (9.6%) needing high-flow nasal cannula oxygen therapy (HFNC). A total of 212 patients (92.5%) were clinically and/or radiologically diagnosed with pneumonia (bilateral in 200 patients, unilateral in 12 patients) at admission. A high-resolution computed tomography (HRCT) of the lungs was performed in 150 (65.6%) patients on admission, and a lung angio-tomography in was performed in 62 (27.0%) patients. Pulmonary embolism was found in six patients (2.6%), while pulmonary congestion was found in nine patients (3.9%) by radiological examination. Nine patients (3.9%) died during hospitalisation.

**Table 1:** Baseline patient characteristics.

Variables	Overall (*n* = 229)
Age (years)	59 (48–67)
Female (*n*, %)	81 (35%)
Male (*n*, %)	148 (65%)
Weight (kg)	86.0 (76.0–97.0)
Height (cm)	172.0 (164.0–178.0)
BMI (kg/m^2^)	29.4 (26.2–32.1)
Waist circumference (cm)	102.0 (93.0–113.0)
Systolic blood pressure (mmHg)	132.0 (120.0–142.0)
Diastolic blood pressure (mmHg)	80.0 (72.0–87.0)
Heart rate (/min)	87.0 (80.0–96.0)
Oxygen saturation (SpO_2_) (%)	91.0 (88.0–94.0)
Comorbidities	
Hypertension (*n*, %)	127 (55.4%)
Obesity (*n*, %)	98 (42.8%)
Diabetes mellitus (*n*, %)	43 (18.8%)
Coronary heart disease (*n*, %)	17 (7.4%)
Atrial fibrillation (*n*, %)	12 (5.2%)
Stroke (*n*, %)	2 (0.9%)
PAD (*n*, %)	3 (1.3%)
COPD (*n*, %)	9 (3.9%)
Asthma (*n*, %)	21 (9.2%)
CKD (*n*, %)	5 (2.2%)
Hypothyreosis (*n*, %)	29 (12.7%)
NPL in the past (*n*, %)	5 (2.2%)
Liver disease (*n*, %)	7 (3.0%)
Depression (*n*, %)	11 (4.8%)
Pharmacotherapy	
ACE inhibitors (*n*, %)	76 (33.2%)
ARB (*n*, %)	29 (12.7%)
Beta-adrenolytics (*n*, %)	70 (30.6%)
Diuretics (*n*, %)	59 (25.8%)
CCB (*n*, %)	56 (24.4%)
MRA (*n*, %)	4 (1.7%)
Alpha-adrenolytics (*n*, %)	19 (8.3%)
Statin (*n*, %)	43 (18.8%)
ASA (*n*, %)	21 (921%)
OAC/NOAC (*n*, %)	8 (3.5%)
Metformin (*n*, %)	41 (17.9%)
SGLT2i (*n*, %)	2 (0.9%)
Sulfonylureas (*n*, %)	15 (6.6%)
Insulin (*n*, %)	4 (1.7%)
GLP-1 agonists (*n*, %)	1 (0.4%)
DPP-4 inhibitors (*n*, %)	2 (0.9%)
Severity of the disease at the admission	
Asymptomatic	6 (2.6%)
Mild illness	20 (8.7%)
Moderate illness	83 (36.2%)
Severe illness	120 (52.4%)
Critical illness	1 (0.4%)
Oxygen therapy	
Nasal cannula	105 (45.8%)
Simple face mask	17 (7.4%)
Non-rebreathing mask	18 (7.9%)
Venturi mask	1 (0.4%)
High-flow nasal cannula	22 (9.6%)
No supplemental oxygen therapy	67 (29.3%)
Biomarkers	
NT-proBNP (pg/ml)	177.0 (71.0–383.0)
Normal (<125 pg/ml)	91 (44.2%)
Mildly elevated (≥125 and <300 pg/ml)	58 (28.1%)
Moderately elevated (≥300 and <1,000 pg/ml)	42 (20.4%)
Significantly elevated (≥1,000 pg/ml)	15 (7.3%)
*Missing data*	*24*
hs-cTnT (ng/ml)	5.9 (3.1–12.0)
Normal (<14 ng/ml)	148 (78.7%)
Elevated (≥14 ng/ml)	40 (21.3%)
*Missing data*	*41*
hs-CRP (mg/L)	85.0 (32.9–126.0)
Outcome	
In-hospital death	9 (3.9%)
ICU	8 (3.5%)
Duration of hospitalisation (days)	13.0 ± 7.7

Italicized values and variable names pertain to a subgroup analysis of the previously presented variable.

Data are presented as *n* (%), mean ± SD, or median (25th–75th percentiles).

BMI, body mass index; PAD, peripheral artery disease; COPD, chronic obstructive pulmonary disease; CKD, chronic kidney disease; NPL, neoplastic disease; ACE, angiotensin-converting enzyme; ARB, angiotensin receptor blocker; CCB, calcium channel antagonist; MRA, mineralocorticoid antagonist; OAC/NOAC, oral anticoagulant/novel oral anticoagulant; hsTnT, high-sensitivity cardiac troponin; CRP, C-reactive protein; ICU, intensive care unit.

Among the associated comorbidities, the most common were found to be arterial hypertension (*n* = 127, 55.4%), obesity (*n* = 98, 42.8%), and diabetes (*n* = 43, 18.8%).

Majority of the clinical and biochemical parameters of the patients admitted to the New University Hospital did not differ significantly from the parameters of the patients admitted to the Temporary COVID Ward, except for higher baseline CRP values [78.0 (35.5–132.5) mg/L vs. 50.9 (24.6–78.5) mg/L; *p* = 0.011] and more frequent need for HFNC therapy (11.3% vs. 2.2%; *p* = 0.065). The data are presented in the Supplementary Table S1.

### 3.2. Baseline echocardiographic characteristics

The left ventricular EF ranged from 47% to 77% (mean 62.7 ± 5.3%). LV was enlarged in nine patients (3.9%). Left ventricular abnormal geometry was found in 104 patients, with the most common concentric LV remodelling (*n* = 66, 28.8%), eccentric hypertrophy was detected in 21 (9.2%) patients, and concentric hypertrophy was noted in 17 patients (7.4%). An increased left atrial volume was observed in 73 patients (31.8%). Nine patients (3.9%) met the present guideline criteria to diagnose diastolic dysfunction (13). The diastolic function was normal in 179 (78.2%) patients, while the diastolic function was classified as undetermined in 41 (17.9%) patients.

An enlargement of the RV was detected in 33 patients (14.4%), an increased right atrial area was found in 90 patients (39.3%), and an increased right atrial volume was observed in 113 patients (49.3%). A decreased RV FAC was present in 29 (12.7%) patients. A reduced TAPSE and decreased *S’* of the tricuspid annulus was present only in two patients (0.8%).

Measuring the LV GLS was possible in 202 patients, and measuring the RV strain was possible in 189 patients. For those patients whom deformation could not be analysed [LV strain in 27 (11.7%), RV strain in 40 (17.5%) patients], the reasons were due to poor image quality, variable heart rate during acquisition, or incomplete acquisition of necessary views. An impaired LV GLS (defined as higher than −18%) was detected in 73 patients (36.1%). The free wall RV strain had decreased (cutoff point −20%) in 111 patients (58.7%). RV thrombus was detected in one patient. Pericardial effusion was present in 11 patients (4.8%). The baseline echocardiographic data are presented in Table 2.

**Table 2:** Baseline echocardiographic parameters.

Echocardiographic parameters	
LVDD (mm)	48.0 (45.0 to 51.0)
LVSD (mm)	32.0 (30.0 to 36.0)
LV EF (%)	63.0 (60.0 to 66.0)
LVEDV (ml)	99.0 (80.0 to 118.0)
LVESV (ml)	36.0 (28.0 to 48.0)
IVSd (mm)	10.0 (9.0 to 12.0)
PWd (mm)	10.0 (9.0 to 11.0)
LVM (g)	169.3 (137.0 to 203.1)
LVMI (g/m^2^)	85.0 (73.0 to 98.2)
RTW	0.41 (0.37 to 0.47)
LV geometry	
Normal	125 (54.6%)
Concentric remodelling	66 (28.8%)
Eccentric hypertrophy	21 (9.2%)
Concentric hypertrophy	17 (7.4%)
LA (mm)	38.0 (35.0 to 41.0)
LAV (ml)	60.0 (50.0 to 70.0)
LAVI (ml/m^2^)	29.6 (25.4 to 35.3)
RVIT (mm)	36.0 (33.5 to 39.0)
RVOT (mm)	30.0 (27.0 to 32.0)
TAPSE (mm)	25.0 (22.0 to 27.0)
S’ (cm/s)	15.0 (13.0 to 17.0)
FAC (%)	43.0 (38.0 to 48.0)
RV d (cm^2^)	19.5 (15.8 to 23.1)
RV s (cm^2^)	11.0 (9.3 to 13.2)
RAV (ml)	46.0 (37.0 to 56.0)
LV GLS (%)	−19.2 (−21.5 to −17.0)
RV FW GLS (%)	−18.7 (−22.6 to −15.0)
LAS-r (%)	28.0 (23.0 to 33.0)
LAS-cd	−14.0 (−18.0 to −11.0)
LAS-ct	−13.0 (−16.0 to −10.0)
TR V max	2.36 (2.16 to 2.59)
LV SV (ml)	72.0 (62.0 to 85.0)
LV CO (L/min)	5.3 (4.4 to 6.2)
E (mm/s)	69.0 (60.0 to 81.0)
A (mm/s)	69.0 (59.0 to 81.0)
E/A	0.97 (0.81 to 1.22)
E’ (mm)	10.0 (8.0 to 12.0)
E/E’	6.8 (6.0 to 8.0)
LVOT V max (m/s)	1.1 (1.0 to 1.2)
RVOT V max (m/s)	0.8 (0.7 to 0.9)
Act (ms)	115.0 (105.0 to 133.0)
Pericardial effusion (*n*, %)	11 (4.8%)
IM (overall; mild/moderate) (*n*, %)	46 (20%); 43 (18.7%)/3 (1.3%)
IT (overall; mild/moderate) (*n*, %)	148 (64.6%); 146 (63.8%)/2 (0.9%)
IA (overall; mild/moderate) (*n*, %)	21 (9.2); 12 (5.2%)/9 (3.9)
IP (overall; mild/moderate) (*n*, %)	19 (8.3%); 18 (7.9%)/1 (0.4%)
Pulmonary hypertension echocardiographic probability (*n*, %)	
Low	116 (50.7%)
Moderate	19 (8.3%)
High	5 (2.2%)
Diastolic function	
Normal	179 (78.2%)
Diastolic dysfunction	9 (3.9%)
Undetermined	41 (17.9%)

Data are presented as *n* (%), mean ± SD, or median (25th–75th percentiles).

LVDD, left ventricular diastolic diameter; LVSD, left ventricular systolic diameter; EF, left ventricular ejection fraction; LVEDV, left ventricular end-diastolic volume; LVESV, left ventricular end-systolic volume; IVSd, interventricular septum diastolic diameter; PWd, posterior wall diastolic diameter; LVM, left ventricular mass; LVMi, left ventricular mass index; RTW, relative wall thickness; LA, left atrium; LAVI, left atrial volume index; RVIT, right ventricular inflow tract diameter; RVOT, right ventricular outflow tract diameter; TAPSE, tricuspid annulus plain systolic excursion; S’, tricuspid annulus systolic velocity; FAC, fractional area change; RVd, right ventricular diastolic area; RVs, right ventricular systolic diameter; RAV, right atrial volume; RAVi right atrial volume index; LVGLS, left ventricular global longitudinal strain; RVFWS, right ventricular free wall strain; LAS-r, left atrial reservoir strain; LAS-cd, left atrial conduit strain; LAS-ct left atrial contraction strain; TR V max, maximal tricuspid regurgitation velocity; LV SV, left ventricular stroke volume; LV CO, left ventricular cardiac output; E, early mitral inflow velocity; A, atrial mitral inflow velocity; E’, mitral annulus early diastolic velocity; LVOT, left ventricular outflow tract; PA Act, pulmonary artery flow acceleration time; IM, mitral regurgitation; IT, tricuspid regurgitation; IA, aortic regurgitation; IP, pulmonic regurgitation.

The interobserver agreement between investigators analysing images was good or very good on overall echocardiographic results, with the highest values for 2D measurements [LVDD: ICC 0.954 (95% CI 0.814–0.995)] and the lowest values for deformation analyses [LAS: ICC 0.801 (95% CI 0.251–0.977)]. The intraobserver variability demonstrated high reproducibility of re-measured parameters [ICC for 24 measurements 0.995 (95% CI 0.986–0.998)].

### 3.3. Cardiac biomarkers

Baseline high-sensitivity cardiac troponin (hs-cTnT) levels were observed in 188 patients, and baseline NT-proBNP levels were observed for 205 patients. Elevated levels of hs-cTnT, that is, values greater than 14 ng/L [>99th percentile upper reference limit (URL)], were observed in 40 (21.3%) patients on admission. More than half of the patients (*n* = 115, 55.8%) at baseline had elevated NT-proBNP levels (i.e., >125 pg/ml; laboratory cutoff point for normal value).

Baseline NT-proBNP level was significantly correlated with indices of the severity of COVID-19, including respiratory rate (*r* = 0.158; *p* = 0.026) and oxygen saturation (SpO_2_) (*r* = −0.450; *p* < 0.001), and parameters of inflammation: hs-CRP H1 (*r* = 0.190; *p* = 0.015), neutrophil count (*r* = 0.287; *p* = −0.016), and lymphocyte count (*r* = −0.187; *p* = 0.016) as well as hs-cTnT (*r* = 0.391; *p* < 0.001) and D-dimers H1 (*r* = 0.301; *p* < 0.001). Among echocardiographic parameters, only NT-proBNP was significantly correlated with LV GLS (*r* = −0.223; *p* = 0.003), RV FAC (*r* = −0.180; *p* = 0.011), TR V max (*r* = 0.381; *p* < 0.001), LA reservoir (*r* = −0.181; *p* = 0.013), and contractile strain (*r* = −0.193; *p* = 0.003).

Cardiac troponin H1, similar to NT-proBNP, was correlated with respiratory rate (*r* = 0.250; *p* = 0.001) and SpO_2_ (*r* = −0.217; *p* = 0.020), while correlation with hs-CRP H1 was of borderline significance (*r* = 0.136; *p* = 0.064). hs-cTnT was also correlated with the diameter of the right ventricle (*r* = 0.250; *p* = 0.032) and negatively correlated with FAC (*r* = −0.26; *p* = 0.028).

Moreover, there was a significant correlation between troponin concentration and end-diastolic interventricular septum (*r* = 0.172; *p* = 0.019) and posterior wall thickness (*r* = 0.198; *p* = 0.007). Myocardial thickness was correlated with hs-cTnT H1 (*r* = 0.198; *p* = 0.007) and SpO_2_ levels (*r* = −0.248; *p* = 0.003), but not with levels of inflammatory markers.

### 3.4. Factors associated with unfavourable course of COVID-19

According to the NIH classification, the initial severe course of the disease was found in 120 patients, with one patient classified as being in a critical condition. In terms of age, patients with a severe/critical disease (*n* = 121) did not differ from patients with an asymptomatic, mild, or moderate disease (total *n* = 109). Despite the lack of differences in the parameters of left ventricular function, significantly higher levels of myocardial necrosis [hs-cTnT H1 7.83 (3.96–15.22) ng/ml vs. 4.97 (2.88–9.37) ng/ml, *p* = 0.01] and markers of heart failure [NT-proBNP 198.0 H1 (93.0–475.0) pg/ml vs. 131.5 (46.3–293.0) pg/ml, *p* = 0.004] were observed in the group of severely ill patients. Patients with a severe course of the disease had lower of SpO_2_ levels [88.0% (85.0%–90.0%) vs. 94.0% (90.0%–96.0%), *p* < 0.001], lower systolic blood pressure (127.9 ± 16.7 vs. 134.8 ± 16.4 mmHg, *p* = 0.002), and lower diastolic blood pressure (78.3 ± 11.4 vs. 82.1 ± 10.4 mmHg, *p* = 0.01). Among echocardiographic parameters, higher TAPSE values [26.0 (IQR 23.0–28.0) mm vs. 24.0 (22.0–27.0) mm; *p* = 0.01], larger right atrial area [17.8 (16.0–20.3) cm^2^ vs. 16.7 (14.7–19.2) cm^2^, *p* = 0.014], and higher *E*/*E’* as a parameter of left ventricular filling pressure [7.09 (6.23–8.28) vs. 6.61 (5.78–7.60), *p* = 0.009] were observed in patients with a severe course of the disease.

Compared with the survivors, the non-survivors were older and had higher respiratory rate and lower SpO_2_ on admission, as well as higher levels of NT-proBNP (both H1 and H7), hs-CRP H7, and D-dimers H7. In the logistic regression analysis, the significant predictors of death were the following: age (*p* = 0.002), hs-CRP H7 (*p* = 0.008), D-dimer H7 (*p* = 0.023), NT-proBNP H7 (*p* < 0.001), tricuspid annulus velocity (*S’*) (*p* = 0.002), TR Vmax (*p* = 0.024), RV FW GLS (*p* = 0.022), and left atrial reservoir strain (*p* = 0.001).

**Figure 2:**
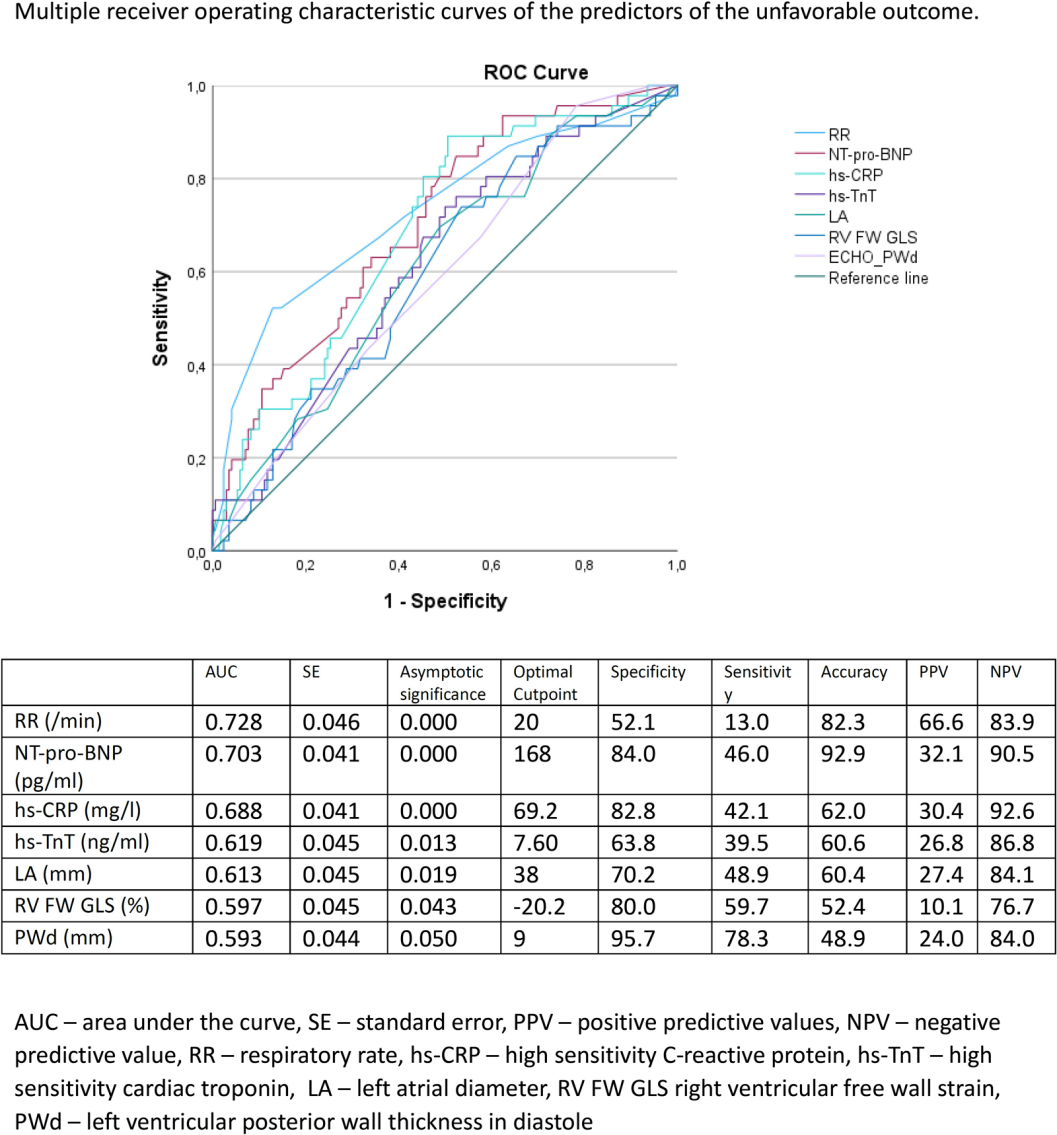
Multiple receiver operating characteristic curves of the predictors of the unfavourable outcome.

An unfavourable outcome was defined as the composite of death (*n* = 9), intensive care unit admission (*n* = 8), or respiratory failure requiring high-flow oxygen therapy (*n* = 44). Patients with unfavourable outcomes were characterised by higher levels of hs-CRP, NT-proBNP, and hs-cTnT (both at baseline and at Day 7). There were no significant differences between the groups in the parameters of LV systolic and diastolic function, while greater left ventricular posterior wall thickness, larger left atrial dimension, and lower left atrial reservoir strain as well as higher TR Vmax and shorter pulmonary artery flow acceleration time were detected (Table 3). Figure 2 presents multiple ROC curves showing the significant predictors of unfavourable outcome and showing the details of the area under the curve, optimal cutpoint, sensitivity, specificity, accuracy, positive predictive value, and negative predictive value of the selected predictors. In the logistic regression analysis, the significant predictors of unfavourable outcome that remained in the model were hs-CRP H1 (*B* = 0.013, *p* = 0.005) and NT-proBNP H1 (*B* = 0.001, *p* = 0.028).

**Table 3:** Clinical, biochemical, and echocardiographic differences between patients with favourable and unfavourable outcome (death, intensive care unit admission, respiratory failure requiring high-flow oxygen therapy).

	Favourable outcome *N* = 182	Unfavourable outcome *N* = 47	*p*
Age (years)	59.0 (47.0 to 67.0)	61.0 (54.0 to 66.0)	0.313
RR (/min)	16.0 (14.0 to 18.0)	20.0 (16.0 to 22.0)	<0.001
SpO_2_ (%)	91.0 (88.0 to 95.0)	88.5 (84.0 to 93.0)	0.032
NT-proBNP (pg/ml)	144.0 (55.75 to 293.0)	348.0 (181.0 to 801.0)	<0.001
hs-cTnT (ng/ml)	5.46 (2.89 to 10.73)	8.44 (4.9 to 15.66)	0.022
hs-CRP_H1 (mg/L)	59.8 (28.85 to 112.5)	107.0 (71.3 to 172.0)	<0.001
PWd (mm)	10.0 (9.0 to 11.0)	10.0 (9.0 to 11.0)	0.031
RWT	0.41 ± 0.07	0.47 ± 0.06	0.007
LA (mm)	37.0 (34.0 to 41)	39.0 (37.0 to 42.0)	0.031
TR V max (m/s)	2.35 ± 0.31	2.48 ± 0.40	0.050
RV FW GLS (%)	−19.2 (−22.8 to −15.1)	−17.2 (−20.1 to −14.0)	0.056
LAS-r (%)	28.0 ± 7.6	25.1 ± 8.5	0.019
Act (ms)	119.0 (105.0 to 136.0)	108.0 (101.0 to 119.0)	0.005

Data are shown as mean ± SD for normally distributed variables and median (ICR) for non-normally distributed variables.

*P*-values for the Student’s *t*-test for independent variables for normally distributed variables and Mann–Whitney *U* test for variables without normal distribution.

RR, respiratory rate; SpO_2_, peripheral blood oxygen saturation; hs-CRP, high-sensitivity C-reactive protein; hs-cTnT, high-sensitivity cardiac troponin; PWd, left ventricular posterior wall thickness in diastole; RTW, relative wall thickness; LA, left atrial diameter; TR Vmax, maximal tricuspid regurgitation velocity; RVFWS, right ventricular free wall strain; LAS-r, left atrial reservoir strain; PA Act, pulmonary artery acceleration time.

### 3.5. Follow-up echocardiography

In the echocardiographic evaluation, the average change in ejection fraction was −1.5% ± 6.7, *p* = 0.008 at the end of the 1-year observation period. An increase of more than 10% in ejection fraction was observed in four patients (1.7%) after 1 year, while a decrease of more than 10% was observed in 21 (9.1%) patients. However, in none of them did EF drop below 45% at the final exam. In the group with an EF decrease of more than 10% in the 1-year follow-up, the patients were characterised by higher left ventricular EF at baseline [68.5% (66.0%–72.0%) vs. 63.0% (60.0%–65.0%), *p* < 0.001], higher levels of TR Vmax (2.5 ± 0.3 m/s vs. 2.3 ± 0.3 m/s; *p* = 0.04), and lower levels of SpO_2_ [88.0% (85.0%–93.0%) vs. 92.0% (89.0%–95.0%), *p* = 0.409].

Features of hyperkinetic circulation were observed in 18 patients from the study group (7.8%) during the first examination, with left ventricular EF of >70%. In the long-term follow-up, EF had decreased to the mean value of 62.7 ± 5.1% (*p* < 0.01). In the group of 14 patients (6.1%) with baseline ejection fraction of <55%, EF was observed to increase from 51.9 ± 2.1% to 57.3 ± 6.4% in the long-term follow-up (*p* < 0.01).

A gradual but significant decrease in myocardial thickness was observed during the 12-month follow-up.

The right ventricular diameter was significantly reduced after 6 months; FAC and pulmonary valve flow acceleration time have improved after 3 months; TAPSE, RW free wall strain, and right atrial volume all had gradually decreased since the first follow-up, as well as the TR Vmax. Improvements in the parameters of left atrial function and early diastolic mitral annulus velocity were also observed. Table 4 presents the echocardiographic parameters assessed at four time points with an analysis of the significance of differences in comparison with the baseline examination.

**Table 4:** Baseline and follow-up echocardiographic characteristics for series of examinations for patients who completed the follow-up (*n* = 168).

	Baseline (1)	3 months (2)	6 months (3)	12 months (4)	*p*
LVDD (mm)	48.0 ± 5.2	47.6 ± 5.3	47.4 ± 4.9	47.8 ± 4.8	0.080
LVSD (mm)	32.8 ± 5.2	31.8 ± 4.8	31.5 ± 5.3^a^	32.7 ± 5.1^c^	0.003
EF (%)	62.9 ± 4.9	62.2 ± 5.9	62.0 ± 5.7	61.6 ± 5.5	0.283
*Baseline EF* ≥*70% (n = 18)*	*72.4 *±* 2.4*	*61.9 *±* 5.*0^a^	*63.4 *±* 6.0*	*62.7 *±* 5.3*^a^	*0*.*003*
*Baseline EF <55% (n = 14)*	*51.9 *±* 2.1*	*52.2 *±* 2.2*	*53.7 *±* 5.5*	*57.3 *±* 8.6*	*0*.*074*
*Baseline EF 55–70 (n = 136)*	*63.1 *±* 3.1*	*62.7 *±* 5.3*	*62.7 *±* 5.3*	*61.8 *±* 6.2*	*0*.*285*
LVEDV (ml)	98.8 ± 29.1	100.6 ± 32.5	98.7 ± 27.9	102.6 ± 30.6	0.200
LVEDV (ml)	38.7 ± 15.5	41.6 ± 16.1	38.5 ± 14.7	40.3 ± 15.6	0.089
IVSd (mm)	10.4 ± 1.6	10.1 ± 2.1	9.8 ± 1.8^a^	9.7 ± 2.0^a^	0.000
PWd (mm)	9.8 ± 1.4	9.4 ± 1.7^a^	9.3 ± 1.4^a^	9.1 ± 1.5^a^	0.000
LVMI (g/m^2^)	87.6 ± 22.2	87.9 ± 25.4	83.1 ± 25.8^a^	78.8 ± 18.8^ab^	0.000
RWT	0.41 ± 0.07	0.40 ± 0.09	0.39 ± 0.07	0.38 ± 0.08^a^	0.000
LAVI (ml/m^2^)	30.5 ± 7.4	29.2 ± 6.9	29.7 ± 7.0	29.8 ± 6.8	0.380
RV 4ch (mm)	35.7 ± 4.6	35.2 ± 4.2	34.7 ± 3.6	34.4 ± 3.6	0.010
RVOT (mm)	29.3 ± 3.8	29.3 ± 3.2	29.5 ± 3.2	29.0 ± 3.3	0.078
TAPSE (mm)	24.4 ± 3.7	23.9 ± 3.9^a^	23.6 ± 3.5^a^	23.5 ± 3.9^a^	0.029
S’ (cm/s)	15.0 ± 2.7	14.9 ± 2.8	14.5 ± 2.6	14.7 ± 2.6	0.592
FAC (%)	42.8 ± 7.6	44.6 ± 6.5^a^	43.7 ± 6.6	44.2 ± 7.0	0.035
RV d (cm^2^)	19.7 ± 5.2	19.5 ± 4.7	19.4 ± 4.7	19.6 ± 4.7	0.922
RV s (cm^2^)	11.5 ± 3.7	11.2 ± 3.5	11.7 ± 5.4	11.4 ± 3.5	0.902
RAV (ml)	46.0 ± 14.8	43.1 ± 14.2^a^	42.1 ± 13.6^a^	41.1 ± 14.0^a^	0.000
LV GLS (%)	−19.1 ± 3.3	−19.2 ± 3.1	−19.9 ± 2.6	−19.7 ± 2.5^a^	0.013
*Baseline GLS* ≤*−18 (n = 40)*	*−15.8 *±* 1.8*	*−18.1 *±* 3.3*^a^	*−19.0 *±* 2.7*^a^	*−18.7 *±* 2.8*^a^	*0*.*000*
*Baseline GLS >−18 (n = 70)*	*−21.0 *±* 2.2*	*19.8 *±* 2.8*^a^	*- 20.4 *±* 2.4*	*−20.3 *±* 2.2*	*0*.*023*
RV FW GLS (%)	−19.9 ± 4.5	−21.5 ± 5.6	−21.8 ± 5.6^a^	−23.2 ± 4.9^a^	0.002
LA strain (%)	29.0 ± 6.8	30.5 ± 6.3	30.1 ± 6.3	29.5 ± 6.0	0.082
LAS-cd	−15.9 ± 6.2	−14.9 ± 6.3	−14.9 ± 5.8	−14.1 ± 5.7	0.055
LAS-ct	−13.1 ± 4.4	−15.6 ± 4.4^a^	−15.2 ± 4.5^a^	−15.6 ± 4.5^a^	0.000
TR V max (m/s)	2.4 ± 0.4	2.2 ± 0.4^a^	2.3 ± 0.4^a^	2.3 ± 0.4^a^	0.047
LV SV (ml)	73.7 ± 19.2	78.3 ± 18.7^a^	78.3 ± 18.6^a^	78.1 ± 17.0^a^	0.015
LV CO (L/min)	5.4 ± 1.5	5.2 ± 1.3	5.0 ± 1.2	5.1 ± 1.2	0.092
HR (/min)	72.6 ± 11.1	65.3 ± 9.0^a^	64.2 ± 9.0^a^	66.1 ± 9.5^a^	0.000
E (cm/s)	69.7 ± 15.8	65.5 ± 15.1^a^	66.5 ± 14.4^a^	65.3 ± 15.2^a^	0.000
A (cm/s)	68.6 ± 15.1	67.3 ± 15.3^a^	65.9 ± 13.6^a^	67.8 ± 14.4^c^	0.017
E/A	1.1 ± 0.3	1.0 ± 0.4^a^	1.1 ± 0.3^b^	1.0 ± 0.3^c^	0.025
E’ (mm)	10.1 ± 2.4	8.7 ± 2.4^a^	9.5 ± 2.4	8.8 ± 2.5^a^	0.000
E/E’	7.1 ± 2.1	8.0 ± 2.3^a^	7.2 ± 2.3^a^	7.2 ± 1.9^a^	0.001
RVOT V max (m/s)	0.8 ± 0.1	0.8 ± 0.1	0.8 ± 0.1	0.9 ± 0.1^ac^	0.003
Act (ms)	120.0 ± 20.0	135.4 ± 23.9^a^	130.4 ± 19.6^a^	129.6 ± 22.2^ab^	0.000

Italicized values and variable names pertain to a subgroup analysis of the previously presented variable.

*P-value refers to* Friedman *one-way* repeated measure analysis *of variance*.

Data are presented as mean ± SD.

LVDD, left ventricular diastolic diameter; LVSD, left ventricular systolic diameter; EF, left ventricular ejection fraction; LVEDV, left ventricular end-diastolic volume; LVESV, left ventricular end-systolic volume; IVSd, interventricular septum diastolic diameter; PWd, posterior wall diastolic diameter; LVM, left ventricular mass; LVMi, left ventricular mass index; RTW, relative wall thickness; LA, left atrium; LAVI, left atrial volume index; RVIT, right ventricular inflow tract diameter; RVOT, right ventricular outflow tract diameter; TAPSE, tricuspid annulus plain systolic excursion; S’, tricuspid annulus systolic velocity; FAC, fractional area change; RVd, right ventricular diastolic area; RVs, right ventricular systolic diameter; RAV, right atrial volume; RAVi right atrial volume index; LVGLS, left ventricular global longitudinal strain; RVFWS, right ventricular free wall strain; LAS-r, left atrial reservoir strain; LAS-cd, left atrial conduit strain; LAS-ct left atrial contraction strain; TR V max, maximal tricuspid regurgitation velocity; LV SV, left ventricular stroke volume; LV CO, left ventricular cardiac output; E, early mitral inflow velocity; A, atrial mitral inflow velocity; E’, mitral annulus early diastolic velocity; LVOT, left ventricular outflow tract; PA Act, pulmonary artery flow acceleration time.

Significant (*p* < 0.05) differences for paired comparisons in Wilcoxon test are marked as follows:

^a^ vs. baseline (2 vs. 1; 3 vs. 1; 4 vs. 1).

^b^ vs. 3-month follow-up (3 vs. 2; 4 vs. 2).

^c^ vs. 12-month follow-up (3 vs. 4).

## 4. Discussion

The main finding of our study is that despite a normal LV systolic function in patients with COVID-19, elevation of cardiac biomarkers is frequent, and this influences in-hospital prognosis. However, it is not followed by a significant impairment of the LV and RV function during the 1-year follow-up. The majority of COVID-19 patients have detectable changes in their cardiac performance in the acute phase of the disease, reflecting an adaptation to an increased haemodynamic stress related to acute inflammatory lung disease.

The increase in the level of cardiac markers correlates with the concentration of inflammatory markers and the severity of the disease as well as the parameters of the pressure load of the right heart chambers. Interestingly, the level of myocardial necrosis markers correlates with myocardial thickness in the acute phase of the disease. In further observation, myocardial thickness gradually decreases, which may indicate the presence of myocardial oedema during the acute phase of COVID-19, suggesting an inflammatory process within the myocardium. However, this does not lead to developing left ventricular systolic dysfunction or heart failure in the 1-year follow-up.

Our observations are in line with those studies documenting the presence of myocardial oedema during the acute phase of COVID-19 in autopsy examinations (15), CMR imaging studies (16), echocardiographic examinations (17), and animal models (18). The myocardial injury in the acute phase of COVID-19 may have complex aetiology and mechanism, including direct damage of cardiomyocytes as well as stress-related injury secondary to elevated cytokines, hypoxaemia, right ventricular strain, and thrombotic complications (3, 19, 20). Interestingly, the echocardiographic study of 43 COVID-19 patients admitted to the intensive care unit revealed that among LV structural abnormalities, an increased myocardial thickness was the most frequent and observed in nearly three-quarters of the patients (17). The authors concluded that the overwhelming cardiac thickness with biventricular pseudohypertrophy indicates a diffuse oedema of the heart. The mechanism of this “swollen heart” phenomenon may possibly be explained by two scenarios—systemic inflammation and cytokine storm, which can lead to a microvascular dysfunction and an increased permeability or by fluid overload during an infection. The concept of COVID-19 as an endothelial disease is now widely accepted (3, 21, 22), and an increased vascular permeability was documented as an effect of SARS-CoV2 infection of *in vitro* cultured endothelial cells (23). The changes in cardiac structure and geometry observed in our study during a 1-year follow-up support the hypothesis of transient myocardial thickening and reversible myocardial oedema in the acute phase of COVID-19. Since inflammatory markers did not show a correlation with myocardial thickness in the studied group, among the mechanisms of this phenomenon, the hypothesis with regard to an increased vascular permeability appears very intriguing. Interestingly, in the COVID-19 animal models, the observations of increased cardiomyocyte size and cardiomyocyte swelling at the peak of viral load were described as a result of the impairment of the pericyte–endothelial crosstalk and the detachment of pericytes from microvasculature (18).

On the other hand, human autopsy studies indicated that acute myocarditis is relatively rare in COVID-19 patients (24, 25), and based on the comprehensive analysis of 22 papers and 277 post-mortem examinations, the most commonly reported cardiac findings are a non-myocarditis inflammatory infiltrate and single-cell ischaemia occurring in 12.6% and 13.7% of cases, respectively (15). Cardiac magnetic resonance (CMR) has the unique capability of characterising myocardial tissue properties *in vivo*, including evaluation for myocardial oedema. Numerous studies have reported CMR abnormalities in patients who recovered from COVID-19 infection (9, 16, 26, 27). A case report was published in the early period of the pandemic presenting an interesting association between elevated biomarkers of myocardial injury with generalised myocardial oedema without late gadolinium enhancement in CMR and normal echocardiogram during COVID-19 (26). Puntmann et al. showed that postinfection features of myocardial inflammations in CMR were present in 60% of 100 patients at a median of 71 days (9). In another study of 148 troponin-positive COVID-19 patients at a median of 68 days, the post-discharge CMR presented a pattern of myocardial inflammation in 26% of the patients, while an ischaemic pattern was detected in 23% (28). Further studies did not confirm those observations (29–31). In a study of 1,285 UK Biobank participants with imaging analysis of pre- and 6-month post-COVID-19 infection, no significant differences in cardiac CMR measures after infection were found in the cases compared with the matched non-infected control group (30). One possible explanation of these discrepancies is the dynamic and potentially reversible characteristic of cardiac involvement in COVID-19, dependent on the time interval between the acute phase of the disease and the observation period and also on comorbidities and preexisting cardiac conditions as well as possibly SARS-CoV2 variant (32). In our study, serial echocardiographic evaluations conducted in patients without structural heart disease revealed that the pattern of left and right heart structure and function changes includes a consistent and gradual decrease in myocardial thickness, left ventricular mass index (LVMi), and relative wall thickness (RTW) as well as an improvement in parameters of the right ventricular and left atrial performance, which were already noticeable in the 3-month follow-up. As the pandemic was developing, numerous studies reported echocardiographic findings in COVID-19 survivors, producing diverging and inconsistent results. Similar with our study, a prospective ECHOVID-19 study including 91 patients revealed that acute COVID-19 negatively affected RV function and cardiac biomarkers with resolution following recovery from disease (33). However, in contrast with our results, the authors observed a reduced LV strain at a baseline examination, which did not improve in the follow-up. Different results were reported by an interesting study retrospectively analysing a series of pre- and post-COVID-19 echocardiograms in the group of 259 individuals (34). When comparing the baseline pre-COVID examinations with post-COVID-19 studies overall no significant differences were found in the left and right ventricular function including EF and GLS and RV free wall strain (FWS). However, a significant worsening of LV GLS was observed in 16 (6.8%) patients, and worsening of RV FWS was detected in 14 (6.0%) patients. Our data correspond to the findings of Young et al. (34) with a similar percentage of patients presenting a decreased LV GLS or RV FWS.

In the World Alliance Societies of Echocardiography COVID study that included 153 patients with paired echocardiograms performed in-hospital and at a median of 129 days of follow-up, LV and RV function was not significantly different; however, some differences in LV and RV function were observed over time according to baseline LV and RV function. In patients with impaired baseline LV or RV function, their performance tended to improve, while it tended to decrease in those with a hyperdynamic LV or normal RV function (35). The same phenomenon indicating a trend of “regression to the mean” was observed in our study population. Patients with higher initial ejection fraction (probably reflecting the adaptation of the circulatory system to the increased metabolic demand resulting from the underlying disease) tended to normalise it, and those with lower ejection fraction tended to improve. Therefore, a statistically significant difference of 1.5% between the baseline and 1-year follow-up can be attributed to the increased EF in the acute phase of the disease rather than its decrease in the follow-up.

The dynamics of changes in cardiac function reflects the haemodynamic load on the heart in the course of acute inflammatory respiratory disease. Many observations in our study exhibit similar echocardiographic and haemodynamic characteristics to those observed in general populations with acute respiratory distress syndrome (ARDS). This raises the question of whether COVID-19-related respiratory failure differs from other ARDS. While studies comparing ARDS with COVID-19 did not yield consistent results, COVID-19 patients with ARDS were found to have lower pulmonary vascular resistance and higher cardiac output compared with ARDS (36–39). The inflammatory and prothrombotic environment associated with COVID-19 is believed to play a role in these observations. However, when it comes to ventilation characteristics and pulmonary haemodynamics, the overall respiratory failure in COVID-19 patients appears to differ only marginally from ARDS.

Enlargement of the right heart chambers, increased tricuspid regurgitation velocity, shortening of pulmonary artery acceleration time, and hypercontractility reflect the homeometric adaptation of the right heart to an increased afterload caused by various factors in respiratory failure and ARDS, such as direct pulmonary endothelial injury/inflammation, microthrombi, and hypoxaemia. Two states of cardiac injury have been described: the first with mild/moderate dilation and compensated function, and the other with RV/LV failure. The initial increase in RV/LV contractility maintains cardiac output, ensuring a sufficient forward flow to meet the oxygen demand. The temporal relationship between these two stages is not clear and may depend on various patient and disease-dependent factors.

In the examined group during the acute phase of the disease, an enlargement of the right atrium was detected in almost half of the population (49.3%), but a decreased RV FAC was observed in only 12.7%. An impairment of RV function was found in over one-half of the patients and an impairment of LV function in one-third of the patients when using more sensitive parameters of ventricular contractility, such as strain.

In our study, reduced RV strain, higher tricuspid valve regurgitation velocity, shorter acceleration time of pulmonary flow, lower LA strain, and increased myocardial thickness were among the factors associated with a worse prognosis.

During the follow-up, changes in the size and function of the heart were observed, including a gradual decrease in heart rate and an increase in stroke volume, as well as improvements in deformation parameters of both ventricles. Furthermore, a reduction in the RV diameter, right atrial volume, and velocity of the regurgitation wave through the tricuspid valve and an increase in the pulmonary flow acceleration time were observed. These consistent changes reflect recovery from the acute phase of the disease and align with the described pathomechanism of alterations associated with acute inflammatory and haemodynamic stress resulting from COVID-19.

In addition, our data demonstrate that assessing left atrial mechanics through strain measurement offers further understanding of cardiac involvement in COVID-19. An acute enlargement and impaired left atrial function may be attributed to increased left ventricular filling pressure and/or direct inflammation-induced atrial myopathy. Previously, reduced left atrial strain has been identified as a predictor of atrial fibrillation and cardiovascular events in the general population (5). In a recently published small study, a reduced left atrial strain was independently associated with the development of long-COVID symptoms (6).

Although our study was an observation of the cohort of homogenous and consecutive patients hospitalised with COVID-19, it was limited by being a single-centre project and by strict inclusion criteria. Focusing on subjects without structural heart disease, as a strategy to avoid the influence of comorbidities on the cardiac performance, resulted in preselection of patients with better prognosis. In our study, the mortality rate was four times lower than in the whole cohort of patients hospitalised in the University Hospital during the pandemic (40), and our results cannot be generalised to the population of hospitalised COVID-19 patients. Even if our study population is modest in size, it is precisely phenotyped and provides unique data on the serial changes of the heart chamber size and function within the 1-year observation. One of the limitations of our study is the fact that although sonographers were blinded to the clinical data of patients and order of examinations, they could not be blinded to the date of the study. We based our observation on echocardiography. In the vast majority of patients, the baseline echo was performed within 72 h from the admission to the hospital. In 19% of the patients, the first echocardiographic examination was performed during the first follow-up visit at the 28th day post-discharge, but there were no significant differences in follow-up changes from the baseline between groups. Although CMR imaging is the most sensitive non-invasive modality to identify and characterise myocardial abnormalities, echocardiography is more practical, available, and most widely used clinically. Interestingly, the serial echocardiograms documented a consistent decrease in myocardial thickness, and since we do not have data on CMR in this population neither any histological examinations, we only speculate on the possibility of myocardial oedema during COVID-19, which however does not lead to cardiac function impairment. Our data support the recommendations that there are no indications for routine echocardiographic control in all patients after COVID-19. Patients without structural heart disease are unlikely to develop heart failure and subclinical left ventricular systolic dysfunction.

In conclusion, in patients hospitalised with COVID-19, an increase in cardiac biomarkers is common and affects in-hospital prognosis. Serial echocardiographic evaluations conducted in patients without preexisting structural heart disease demonstrate an overall trend towards an improved biventricular function and a reduced myocardial thickening during the 1-year follow-up. This suggests that in patients without preexisting conditions, the acute cardiac consequences of COVID-19 are associated with systemic inflammation and haemodynamic stress. However, even in patients with a severe course of the disease, no clinically significant cardiac dysfunction is observed after 1 year.

## Data Availability

The raw data supporting the conclusions of this article will be made available by the authors, without undue reservation.

## References

[1] Huang C Wang Y Li X Ren L Zhao J Hu Y Clinical features of patients infected with 2019 novel coronavirus in Wuhan, China. Lancet. (2020) 395(10223):497–506. 10.1016/S0140-6736(20)30183-5 31986264 PMC7159299

[2] Ruan Q Yang K Wang W Jiang L Song J. Clinical predictors of mortality due to COVID-19 based on an analysis of data of 150 patients from Wuhan, China. Intensive Care Med. (2020) 46(5):846–8. 10.1007/s00134-020-05991-x 32125452 PMC7080116

[3] Guzik TJ Mohiddin SA Dimarco A Patel V Savvatis K Marelli-Berg FM COVID-19 and the cardiovascular system: implications for risk assessment, diagnosis, and treatment options. Cardiovasc Res. (2020) 116(10):1666–87. 10.1093/cvr/cvaa106 32352535 PMC7197627

[4] Zhou F Yu T Du R Fan G Liu Y Liu Z Clinical course and risk factors for mortality of adult inpatients with COVID-19 in Wuhan, China: a retrospective cohort study. Lancet. (2020) 395(10229):1054–62. 10.1016/S0140-6736(20)30566-3 32171076 PMC7270627

[5] Shi S Qin M Shen B Cai Y Liu T Yang F Association of cardiac injury with mortality in hospitalized patients with COVID-19 in Wuhan, China. JAMA Cardiol. (2020) 5(7):802–10. 10.1001/jamacardio.2020.0950 32211816 PMC7097841

[6] Lippi G Lavie CJ Sanchis-Gomar F. Cardiac troponin I in patients with coronavirus disease 2019 (COVID-19): evidence from a meta-analysis. Prog Cardiovasc Dis. (2020) 63(3):390–1. 10.1016/j.pcad.2020.03.001 32169400 PMC7127395

[7] Cunningham JW Claggett BL Jering KS Vaduganathan M Bhatt AS Rosenthal N Prognostic value of natriuretic peptides and cardiac troponins in COVID-19. Circulation. (2021) 144(2):177–9. 10.1161/CIRCULATIONAHA.121.054969 33999648 PMC8270227

[8] Huang S Vignon P Mekontso-Dessap A Tran S Prat G Chew M Echocardiography findings in COVID-19 patients admitted to intensive care units: a multi-national observational study (the ECHO-COVID study). Intensive Care Med. (2022) 48(6):667–78. 10.1007/s00134-022-06685-2 35445822 PMC9022062

[9] Puntmann VO Carerj ML Wieters I Fahim M Arendt C Hoffmann J Outcomes of cardiovascular magnetic resonance imaging in patients recently recovered from coronavirus disease 2019 (COVID-19). JAMA Cardiol. (2020) 5(11):1265–73. 10.1001/jamacardio.2020.3557 32730619 PMC7385689

[10] Sydor W Wizner B Strach M Bociąga-Jasik M Mydel K Olszanecka A CRACoV-HHS: an interdisciplinary project for multi-specialist hospital and non-hospital care for patients with SARS-CoV-2 infection as well hospital staff assessment for infection exposure. Folia Med Cracov. (2021) 61(4):5–44. 10.24425/fmc.2021.140002 35180200

[11] Coronavirus disease 2019 (COVID-19) treatment guidelines [Internet]. Bethesda, MD: National Institutes of Health (US) (2021) Apr 21-2023 Jul 21. Available from: https://www.ncbi.nlm.nih.gov/books/NBK570371/34003615

[12] Lang RM Badano LP Mor-Avi V Afilalo J Armstrong A Ernande L Recommendations for cardiac chamber quantification by echocardiography in adults: an update from the American Society of Echocardiography and the European Association of Cardiovascular Imaging. J Am Soc Echocardiogr. (2015) 28(1):1–39.e14. 10.1016/j.echo.2014.10.003 25559473

[13] Nagueh SF Smiseth OA Appleton CP Byrd BF 3rd Dokainish H Edvardsen T Recommendations for the evaluation of left ventricular diastolic function by echocardiography: an update from the American Society of Echocardiography and the European Association of Cardiovascular Imaging. Eur Heart J Cardiovasc Imaging. (2016) 17(12):1321–60. 10.1093/ehjci/jew082 27422899

[14] Humbert M Kovacs G Hoeper MM Badagliacca R Berger RMF Brida M 2022 ESC/ERS guidelines for the diagnosis and treatment of pulmonary hypertension: developed by the task force for the diagnosis and treatment of pulmonary hypertension of the European Society of Cardiology (ESC) and the European Respiratory Society (ERS). Endorsed by the International Society for Heart and Lung Transplantation (ISHLT) and the European Reference Network on Rare Respiratory Diseases (ERN-LUNG). Eur Heart J. (2022) 43(38):3618–731. 10.1093/eurheartj/ehac237 36017548

[15] Halushka MK Vander Heide RS. Myocarditis is rare in COVID-19 autopsies: cardiovascular findings across 277 postmortem examinations. Cardiovasc Pathol. (2021) 50:107300. 10.1016/j.carpath.2020.107300 33132119 PMC7583586

[16] Petersen SE Friedrich MG Leiner T Elias MD Ferreira VM Fenski M Cardiovascular magnetic resonance for patients with COVID-19. JACC Cardiovasc Imaging. (2022) 15(4):685–99. 10.1016/j.jcmg.2021.08.021 34656482 PMC8514168

[17] Liu Y Xie J Gao P Tian R Qian H Guo F Swollen heart in COVID-19 patients who progress to critical illness: a perspective from echo-cardiologists. ESC Heart Fail. (2020) 7(6):3621–32. 10.1002/ehf2.12873 32977359 PMC7646648

[18] Daems M Liesenborghs L Boudewijns R Simmonds SJ Kraisin S Van Wauwe J SARS-CoV-2 infection causes prolonged cardiomyocyte swelling and inhibition of HIF1α translocation in an animal model COVID-19. Front Cardiovasc Med. (2022) 9:964512. 10.3389/fcvm.2022.964512 36324747 PMC9618878

[19] Basso C Leone O Rizzo S De Gaspari M van der Wal AC Aubry MC Pathological features of COVID-19-associated myocardial injury: a multicentre cardiovascular pathology study. Eur Heart J. (2020) 41(39):3827–35. 10.1093/eurheartj/ehaa664 32968776 PMC7543528

[20] Xu SC Wu W Zhang SY. Manifestations and mechanism of SARS-CoV2 mediated cardiac injury. Int J Biol Sci. (2022) 18(7):2703–13. 10.7150/ijbs.69677 35541905 PMC9066113

[21] Pelisek J Reutersberg B Greber UF Zimmermann A. Vascular dysfunction in COVID-19 patients: update on SARS-CoV-2 infection of endothelial cells and the role of long non-coding RNAs. Clin Sci. (2022) 136(21):1571–90. 10.1042/CS20220235 PMC9652506 36367091

[22] Libby P Lüscher T. COVID-19 is, in the end, an endothelial disease. Eur Heart J. (2020) 41(32):3038–44. 10.1093/eurheartj/ehaa623 32882706 PMC7470753

[23] Rauti R Shahoha M Leichtmann-Bardoogo Y Nasser R Paz E Tamir R Effect of SARS-CoV-2 proteins on vascular permeability. eLife. (2021) 10:e69314. 10.7554/eLife.69314 34694226 PMC8545399

[24] Almamlouk R Kashour T Obeidat S Bois MC Maleszewski JJ Omrani OA COVID-19-associated cardiac pathology at the postmortem evaluation: a collaborative systematic review. Clin Microbiol Infect. (2022) 28(8):1066–75. 10.1016/j.cmi.2022.03.021 35339672 PMC8941843

[25] Lindner D Fitzek A Bräuninger H Aleshcheva G Edler C Meissner K Association of cardiac infection with SARS-CoV-2 in confirmed COVID-19 autopsy cases. JAMA Cardiol. (2020) 5(11):1281–5. 10.1001/jamacardio.2020.3551 32730555 PMC7385672

[26] Manka R Karolyi M Polacin M Holy EW Nemeth J Steiger P Myocardial edema in COVID-19 on cardiac MRI. J Heart Lung Transplant. (2020) 39(7):730–2. 10.1016/j.healun.2020.04.025 32650881 PMC7834291

[27] Ruberg FL Baggish AL Hays AG Jerosch-Herold M Kim J Ordovas KG Utilization of cardiovascular magnetic resonance imaging for resumption of athletic activities following COVID-19 infection: an expert consensus document on behalf of the American Heart Association Council on Cardiovascular Radiology and Intervention Leadership and endorsed by the Society for Cardiovascular Magnetic Resonance. Circ Cardiovasc Imaging. (2023) 16(1):e014106. 10.1161/CIRCIMAGING.122.014106 36541203 PMC9848221

[28] Kotecha T Knight DS Razvi Y Kumar K Vimalesvaran K Thornton G Patterns of myocardial injury in recovered troponin-positive COVID-19 patients assessed by cardiovascular magnetic resonance. Eur Heart J. (2021) 42(19):1866–78. 10.1093/eurheartj/ehab075 33596594 PMC7928984

[29] Joy G Artico J Kurdi H Seraphim A Lau C Thornton GD Prospective case-control study of cardiovascular abnormalities 6 months following mild COVID-19 in healthcare workers. JACC Cardiovasc Imaging. (2021) 14(11):2155–66. 10.1016/j.jcmg.2021.04.011 33975819 PMC8105493

[30] Bai W Raman B Petersen SE Neubauer S Raisi-Estabragh Z Aung N Longitudinal changes of cardiac and aortic imaging phenotypes following COVID-19 in the UK Biobank cohort. medRxiv. (2021) 11.04.21265918. 10.1101/2021.11.04.21265918

[31] Singh T Kite TA Joshi SS Spath NB Kershaw L Baker A MRI and CT coronary angiography in survivors of COVID-19. Heart. (2022) 108(1):46–53. 10.1136/heartjnl-2021-319926 34615668 PMC8503921

[32] Ghantous E Shetrit A Hochstadt A Banai A Lupu L Levi E Cardiologic manifestations in omicron-type versus wild-type COVID-19: a systematic echocardiographic study. J Am Heart Assoc. (2023) 12(3):e027188. 10.1161/JAHA.122.027188 36695308 PMC9973649

[33] Lassen MCH Skaarup KG Lind JN Alhakak AS Sengeløv M Nielsen AB Recovery of cardiac function following COVID-19 - ECHOVID-19: a prospective longitudinal cohort study. Eur J Heart Fail. (2021) 23(11):1903–12. 10.1002/ejhf.2347 34514713 PMC8652600

[34] Young KA Krishna H Jain V Hamza I Scott CG Pellikka PA Serial left and right ventricular strain analysis in patients recovered from COVID-19. J Am Soc Echocardiogr. (2022) 35(10):1055–63. 10.1016/j.echo.2022.06.007 35760277 PMC9232260

[35] Karagodin I Singulane CC Descamps T Woodward GM Xie M Tucay ES Ventricular changes in patients with acute COVID-19 infection: follow-up of the world alliance societies of echocardiography (WASE-COVID) study. J Am Soc Echocardiogr. (2022) 35(3):295–304. 10.1016/j.echo.2021.10.015 34752928 PMC8572036

[36] Caravita S Baratto C Di Marco F Calabrese A Balestrieri G Russo F Haemodynamic characteristics of COVID-19 patients with acute respiratory distress syndrome requiring mechanical ventilation. An invasive assessment using right heart catheterization. Eur J Heart Fail. (2020) 22(12):2228–37. 10.1002/ejhf.2058 33200458 PMC7753704

[37] Becker A Seiler F Muellenbach RM Danziger G Kamphorst M Lotz C Pulmonary hemodynamics and ventilation in patients with COVID-19-related respiratory failure and ARDS. J Intensive Care Med. (2021) 36(6):655–63. 10.1177/0885066621995386 33678052

[38] Haudebourg AF Perier F Tuffet S de Prost N Razazi K Mekontso Dessap A Respiratory mechanics of COVID-19- versus non-COVID-19-associated acute respiratory distress syndrome. Am J Respir Crit Care Med. (2020) 202(2):287–90. 10.1164/rccm.202004-1226LE 32479162 PMC7365370

[39] Gattinoni L Coppola S Cressoni M Busana M Rossi S Chiumello D. COVID-19 does not lead to a “typical” acute respiratory distress syndrome. Am J Respir Crit Care Med. (2020) 201(10):1299–300. 10.1164/rccm.202003-0817LE 32228035 PMC7233352

[40] Wojciechowska W Terlecki M Klocek M Pac A Olszanecka A Stolarz-Skrzypek K Impact of arterial hypertension and use of antihypertensive pharmacotherapy on mortality in patients hospitalized due to COVID-19: the CRACoV-HHS study. Hypertension. (2022) 79:2601–10. 10.1161/hypertensionaha.122.19575 36082666 PMC9553221

