# Supplementary material for: Serial echocardiographic evaluation of COVID-19 patients without prior history of structural heart disease: a 1-year follow-up CRACoV-HHS study
Source: Front Cardiovasc Med. 2023 Sep 13;10:1230669. doi: 10.3389/fcvm.2023.1230669 (PMC10533911; doi:10.3389/fcvm.2023.1230669)
Supplement: Supplementary file 1 [file Datasheet1.docx]

**Supplementary materials**

**Serial echocardiographic evaluation of COVID-19 patients without previous history of structural heart disease – one year follow-up CRACoV-HHS study**

**Agnieszka Olszanecka*^1,2^, Wiktoria Wojciechowska^1,2^, Agnieszka Bednarek^2^, Piotr Kusak^2^, Barbara Wizner^3^, Michał Terlecki^1,2^, Katarzyna Stolarz-Skrzypek^1,2^, Marek Klocek^1,2^, Tomasz Drożdż^1,2^, Krzysztof Sładek^4,2^, Monika Bociąga-Jasik^5,2^, Aleksander Garlicki^5,2^, Krzysztof Rewiuk^3,2^, Andrzej Matyja^6,2^, Maciej Małecki^7,2^, Wojciech Sydor^8, 2^, Marcin Krzanowski^9,2^, Tomasz Grodzicki^3,2^, Marek Rajzer^1,2^ and CRACoV-HHS Investigators†**

*** Correspondence:**Agnieszka Olszanecka, MD, PhD
agnieszka.olszanecka@uj.edu.pl

†The CRACoV-HHS Study Investigators

Adamczyk Michalina, Andrychiewicz Anna, Antczak Jakub, Banaszkiewicz Małgorzata, Barańska Ilona, Barbara Żółtowska, Bartuś Stanisław, Bednarek Agnieszka, Bednarek Joanna, Bętkowska -Korpała Barbara, Bień Artur Igor, Bociąga-Jasik Monika, Brandt Łukasz, Brudło Michał, Bryll Amira, Bryniarski Leszek, Bryniarski Paweł, Brzychczy Barbara, Brzychczy-Włoch Monika, Bugajski Janusz, Bujak- Giżycka Beata, Burliga Tomasz, Celejewska-Wojcik Natalia, Chatys-Bogacka Żaneta, Cholewczuk Agnieszka, Chromik- Legień Anna, Chrzan Robert, Chyrchel Bernadeta, Chyrchel Michał, Ciesielska Kinga, Cyranka Katarzyna, Czaikivska Zlata, Czepiel Jacek, Czepiel Klaudia, Czyżycki Mateusz, Ćwięk Aleksandra, Dembe Katarzyna, Dembiński Marcin, Drożdż Tomasz, Dudek Aleksandra, Dudek Dominika, Dwojak Mateusz, Dziewierz Artur, Dzieża-Grudnik Anna, Fedyk-Łukasik Małgorzata, Fiema Mateusz, Furman Katarzyna, Gacek Magdalena, Gajda Mateusz, Garlicki Aleksander, Garlicki Jarosław, Gąsowski Jerzy, Gołasa Paulina, Gosiewski Tomasz, Górka Karolina, Gradek-Kwinta Elżbieta, Gregorczyk-Maga Iwona, Grodzicki Tomasz, Gross-Sondej Iwona, Gryglewska Barbara, Hajek Agnes, Hartwich Patryk, Hohendorff Jerzy, Huras Hubert, Jachowicz Estera, Jagiełła Jeremiasz, Jagiełło Wojciech, Kamińska Barbara, Kania Aleksander, Kania Michał, Kapusta Przemysław, Karcz Paulina, Kasper Łukasz, Kasprzycki Karol, Kasprzyk Jakub, Katra Barbara, Kędzierska Jolanta, Kępińska-Wnuk Alicja, Kęsek Tomasz, Kiepura Anna, Kijowska Violetta, Klocek Marek, Klupa Tomasz, Kołak Magdalena, Kopeć Jolanta, Kopka Marianna, Kostrzycka Małgorzata, Kowina Natalia, Koźmiński Wojciech, Krawczyk Jacek, Krzanowska Katarzyna, Krzanowski Marcin, Krzyściak Paweł, Krzyściak Wirginia, Kukla Michał, Kusak Piotr, Laskowska - Wronarowicz Anna, Lechowicz Patrycja, Liberacka Donata, Lichołai Sabina, Lickiewicz Beata, Lorkowska- Zawicka Barbara, Łomnicka Magdalena, Łukasik Stanisław, Mach Krzysztof, Madej Józef, Majka Wojciech, Major Piotr, Małecki Maciej, Marona Monika, Matyja Andrzej, Maziarz Barbara, Mazur Konad,Mazurkiewicz Iwona, Motyl Maciej, Mrugacz Marcin, Mydel Krzysztof, Nastałek Paweł, Noga Magdalena, Nowak Klaudia, Nowakowski Michał, Olszanecka Agnieszka, Olszanecki Rafał, Olszewska - Turek Katarzyna, Ostrowski Wojciech, Pałczyńska Ewa, Pałka Anna, Pastuszak - Draxler Anna, Pawela Małgorzata, Pawliński Łukasz, Perera Ian, Petkow-Dimitrow Paweł, Pędziwiatr Michał, Piątek Anna, Piętak Ewelina, Pilecki Maciej, Piotrowicz Karolina, Podolski Adrian, Polok Kamil, Popiela Tadeusz, Pośpiech Kamila, Przybyszowski Marek, Puchalska Karolina, Pułyk Agnieszka, Pyrć Krzysztof, Pytel Krzysztof, Rajzer Marek, Rakowski Tomasz, Rojek-Zakrzewska Danuta, Romaniszyn Dorota, Różańska Anna, Rudnik Gabriela, Rudzki Łukasz, Rybicka Monika, Rymarowicz Justyna, Rzemińska Agnieszka, Rzeszutko Łukasz, Rzeźnik Monika, Salamon Dominika, Sanak Marek, Sarna-Palacz Dominika, Sawczyńska Katarzyna, Sepioło Anna, Sewiło Jakub, Siwiec Andżelika, Skalska Małgorzata, Skalska-Świstek Małgorzata, Skóra Magdalena, Sładek Krzysztof , Słowik Agnieszka , Sroka-Oleksiak Agnieszka, Stachowicz Aneta, Stachura Tomasz, Starowicz - Filip Anna, Starzyk Malwina, Stolarz-Skrzypek Katarzyna, Strach Magdalena, Struś Michał, Sulicka-Grodzicka Joanna, Surdacki Andrzej, Surowiec Paulina, Suski Maciej, Sydor Wojciech, Szaleniec Joanna, Szczerbińska Katarzyna, Szwajca Marta, Śmierciak Natalia, Talaga- Ćwiertnia Katarzyna, Terlecki Michał, Tokarczyk Zuzanna, Tomik Jerzy, Totoń- Żurańska Justyna, Trojan-Królikowska Anna, Turek Aleksander, Ucieklak Damian, Urbanik Andrzej, Walczewska Jolanta, Wężyk Kamil, Widera Alicja, Wierdak Mateusz, Wilk Magdalena, Winiarski Marek Witek Przemysław, Wizner Barbara, Włodarczyk Małgorzata, Wnuk Marcin, Wojciechowska Wiktoria, Wojkowska-Mach Jadwiga, Woroń Jarosław, Woziwodzka Karolina, Wójcik Krzysztof, Wójkowska-Mach Jadwiga, Wrona Paweł, Zarzecka -Francica Elżbieta, Zarzecka Joanna, Zawadzka Barbara, Zięba-Parkitny Joanna, Żurowicz Bożena
