# Supplementary material for: Serial echocardiographic evaluation of COVID-19 patients without prior history of structural heart disease: a 1-year follow-up CRACoV-HHS study
Source: Front Cardiovasc Med. 2023 Sep 13;10:1230669. doi: 10.3389/fcvm.2023.1230669 (PMC10533911; doi:10.3389/fcvm.2023.1230669)
Supplement: Supplementary file 2 [file Table1.docx]

Suplementary table 1.

**Table 1.** Baseline patients characteristics of patients hospitalized in the New University Hospital and Temporal Covid Ward.

|  | NUH  n=184 | TCW  n=45 | p |
| --- | --- | --- | --- |
| Age (years) | 59 (50; 66) | 61 (47; 68) | 0.728 |
| Female (n, %) | 65 (35%) | 16 (36%) | 0.929 |
| Male (n, %) | 119 (65%) | 29 (64% | 0.968 |
| Weight (kg) | 87.0 (77.5-97) | 83.0 (73.0-97.0) | 0.201 |
| Height (cm) | 173.0 (164.0-178.0) | 170.0 (162.0- 176.0) | 0.150 |
| BMI (kg/m2) | 29.4 (26.2-32.2)) | 29.0 (26.6-31.9) | 0.545 |
| Waist circumference (cm) | 102.0 (93.0-112.0) | 105.5 (92.0- 115) | 0.841 |
| Systolic blood pressure (mmHg) | 131.0 (120.0-140.0) | 135.0 (122.5- 147.0) | 0.160 |
| Diastolic blood pressure (mmHg) | 80.0 (72.0-87.0) | 82.0 (77.0- 87.5) | 0.307 |
| Heart rate (/min) | 88.0 (80.0- 96.0) | 86.0 (75.5- 96.0) | 0.446 |
| Oxygen saturation (SpO2) (%) | 91.0 (88.0- 95.0) | 93.0 (89.0- 94.0) | 0.274 |
| Comorbidities | | | |
| Hypertension (n, %) | 102 (55.1%) | 25 (55.5%) | 0.927 |
| Obesity (n, %) | 81 (44.0%) | 17 (38.6%) | 0.678 |
| Diabetes mellitus (n, %) | 37 (20.0%) | 6 (13.3%) | 0.407 |
| Coronary heart disease (n, %) | 13 (7.0%) | 4 (8.9%) | 0.672 |
| Atrial fibrillation (n, %) | 12 (6.4%) | 0 (0%) | 0.092 |
| Stroke (n, %) | 1 (0.5%) | 1 (2.2%) | 0.275 |
| PAD (n, %) | 3 (1.5%) | 0 (0%) | 0.397 |
| COPD (n, %) | 5 (2.7%) | 4 (8.8% | 0.085 |
| Asthma (n, %) | 16 (8.6%) | 5 (11.1%) | 0.618 |
| CKD (n, %) | 5 (2.7%) | 0 (0%) | 0.275 |
| NPL in the past (n, %) | 5 (2.7%) | 0 (0%) | 0.275 |
| Liver disease (n, %) | 5 (2.7%) | 2 (4.4%) | 0.5426 |
| Depression (n, %) | 9 (4.9%) | 2 (4.4%) | 0.926 |
|  | | | |
| ACE inhibitors (n,%) | 64 (34.5%) | 12 (26.7%) | 0.494 |
| ARB (n,%) | 22 (11.9%) | 7 (15.6%) | 0.538 |
| Beta-adrenolytics (n, %) | 62 (33.5%) | 8 (17.8%) | 0.129 |
| Diuretics (n,%) | 48 (25.9%) | 11 (24.4%) | 0.909 |
| CCB (n, %) | 47 (25.4%) | 9 (20.0%) | 0.578 |
| MRA (n, %) | 2 (1.1%) | 2 (4.4%) | 0.128 |
| Alpha-adrenolytics (n,%) | 18 (9.7%) | 1 (2.2%) | 0.127 |
| Statin (n, %) | 39 (21.0%) | 4 (8.9) | 0.115 |
| ASA (n, %) | 18 (9.7%) | 3 (6.7%) | 0.578 |
| OAC/NOAC (n, %) | 7 (3.8%) | 1 (2.2%) | 0.630 |
| Metformin (n, %) | 35 (18.9%) | 6 (13.3%) | 0.479 |
| SGLT2i (n, %) | 2 (1.1%) | 0 (0%) | 0.489 |
| Sulfonylureas (n %) | 13 (7.0%) | 2 (4.4%) | 0.567 |
| Insulin (n, %) | 4 (2.1%) | 0 (0%) | 0.329 |
| GLP-1 agonists (n, %) | 1 (0.5%) | 0 (0%) | 0.624 |
| DPP-4 inhibitors (n,%) | 2 (1.1%) | 0 (0%) | 0.489 |
|  | | | |
| Asymptomatic | 6 (3.2%) | 0 (0%) | 0.232 |
| Mild illness | 18 (9.7%) | 2 (4.4%) | 0.305 |
| Moderate illness | 63 (28.6%) | 20 (44.4%) | 0.354 |
| Severe illness | 97 (52.4%) | 23 (51.2%) | 0.976 |
| Critical illness | 1 (0.5%) | 0 (0%) | 0.624 |
| Oxygen therapy | | | |
| Nasal cannula | 83 (44.9%) | 22 (48.9) | 0.694 |
| Simple face mask | 13 (7.0%) | 4 (8.9%) | 0.671 |
| Non-rebreathing mask | 15 (8.1%) | 3 (6.7%) | 0.784 |
| Venturi mask | 1 (0.5%) | 0 (0%) | 0.625 |
| High-flow nasal cannula | 21 (11.3%) | 1 (2.2%) | 0.065 |
| No supplemental oxygen therapy | 52 (28.2%) | 15 (33.3%) | 0.578 |
| Biomarkers | | | |
| NT-pro-BNP (ng/ml) | 179.0 (76.0- 415.0) | 169.0 (46.0- 262.0) | 0.197 |
| normal (<125 ng/ml) | 68 (42.1%) | 22 (52.4%) | 0.331 |
| mildly elevated (≥125 and <300 ng/ml) | 46 (28.0%) | 12 (28.6%) | 0.811 |
| moderately elevated (≥300 and <1000 ng/ml) | 35 (21.3%) | 7 (16.7%) | 0.688 |
| significantly elevated (≥ 1000 ng/ml) | 14 (8.6%) | 1 (2.3%) | 0.222 |
| *missing data* | *21* | *3* | 0.416 |
| hsTnT (ng/ml) |  |  | 0.842 |
| normal (<14 ng/ml) | 112 (77.9%) | 35 (79.6%) | 0.317 |
| elevated (≥ 14 ng/ml) | 32 (22.1%) | 9 (20.4%) | 0.694 |
| *missing data* | *40* | *1* | 0.007 |
| CRP (ng/ml) | 78.0 (35.5- 132.5) | 50.9 (24.6- 78.5) | 0.011 |
| Outcome |  |  |  |
| In-hospital death | 9 (4.8%) | 0 (0%) | 0.144 |
| ICU | 8 (4.3%) | 0 (0%) | 0.168 |
| duration of hospitalization (days) | 11.0 (9.0- 15.0) | 11.0 (9.0- 14.) | 0.665 |

Data are presented as n(%) and median (25^th^-75^th^ percentiles).

P value refers to the student t-test or the Mann-Whitney U test for non-normally distributed continuous variables. Categorical variables were compared based on chi-square testing or Fisher’s exact test.

BMI – body mass index, PAD – peripheral artery disease, COPD – chronic obstructive pulmonary disease, CKD – chronic kidney disease, NPL – neoplastic disease, ACE – angiotensin converting enzyme, ARB – angiotensin receptor blocker, CCB – calcium channel antagonist, MRA – mineralocorticoid antagonist, OAC/NOAC – oral anticoagulant/novel oral anticoagulant, hsTnT – high sensitivity cardiac troponin, CRP – C-reactive protein, ICU – intensive care unit
